# Municipalities' Preparedness for Weather Hazards and Response to Weather Warnings

**DOI:** 10.1371/journal.pone.0163390

**Published:** 2016-09-20

**Authors:** Kaddour Mehiriz, Pierre Gosselin

**Affiliations:** 1 Centre Eau, Terre et Environnement, Institut National de la Recherche Scientifique, Québec, Québec, Canada; 2 Institut National de la Santé Publique du Québec, Québec, Québec, Canada; 3 OURANOS, Montréal, Québec, Canada; Centro de Investigacion Cientifica y de Educacion Superior de Ensenada Division de Fisica Aplicada, MEXICO

## Abstract

The study of the management of weather-related disaster risks by municipalities has attracted little attention even though these organizations play a key role in protecting the population from extreme meteorological conditions. This article contributes to filling this gap with new evidence on the level and determinants of Quebec municipalities’ preparedness for weather hazards and response to related weather warnings. Using survey data from municipal emergency management coordinators and secondary data on the financial and demographic characteristics of municipalities, the study shows that most Quebec municipalities are sufficiently prepared for weather hazards and undertake measures to protect the population when informed of imminent extreme weather events. Significant differences between municipalities were noted though. Specifically, the level of preparedness was positively correlated with the municipalities’ capacity and population support for weather-related disaster management policies. In addition, the risk of weather-related disasters increases the preparedness level through its effect on population support. We also found that the response to weather warnings depended on the risk of weather-related disasters, the preparedness level and the quality of weather warnings. These results highlight areas for improvement in the context of increasing frequency and/or severity of such events with current climate change.

## Introduction

The organization of emergency management varies according to the countries’ political and administrative systems though a general consensus exists according to which the majority of weather-related disaster risks are of local nature and thus the active involvement of local governments is needed to effectively reduce them [1, 2]. The decentralization of the management of weather-related disaster risks is presumed to improve the accountability and transparency, the participation of citizens in the decision making and, ultimately, to match civil protection services with the needs of local communities and their capacity to pay [3]. At the same time, it raises serious questions about whether local governments have the financial and administrative capacity and political incentives to reduce the risk of weather-related disasters [3, 4].

Although municipalities are key stakeholders in the management of weather disaster risks, empirical studies on their activities in this field remain scarce [5] and, the majority of them use qualitative methods that impose tight constraints on the possibility to generalize findings to other contexts (see [6, 7] among others). This article aims to reduce the gap of knowledge on this topic as it reports the results of a quantitative study on the management of weather-related disaster risks by Quebec municipalities. Specifically, survey data from municipal emergency management coordinators and administrative records are used to provide responses to the following questions:

1. How are municipalities prepared for weather-related disasters?
2. How do they respond to weather warnings?
3. What are the factors that explain their levels of preparedness for weather-related disasters and responses to weather warnings?

## The Organization of Weather-Related Disaster Risk Management System in Quebec-Canada

Canadian provinces are responsible for weather-related disaster risk management in their respective territories, whereas the federal government operates in its exclusive fields of jurisdiction and on the lands and properties under federal responsibility [8]. The federal government may, however, respond to any request for assistance from a provincial government.

In Quebec, emergency management comes under the Civil Protection Act of Quebec (*Loi sur la sécurité civile* (L.R.Q., C. S-2.3) that provides for a sharing of responsibility between the government, municipalities, citizens and businesses. The Government of Quebec’s role consists in planning and coordinating provincial emergency measures and in providing support for municipalities dealing with disasters of any kind that are beyond their capacity. To fulfil its mission, the Quebec Government developed provincial and regional emergency management plans and assigned the coordination of public organizations involved in their implementation to the Quebec Department of Public Security (QDPS).

As is the case in the other Canadian provinces [9], the Government of Quebec has delegated a large portion of its emergency management prerogatives to municipalities but has included mechanisms to monitor their activities. The Civil Protection Act of Quebec requires that regional authorities, namely, the regional county municipalities (MRC), large cities and any municipality designated by the QDPS, develop, in collaboration with the municipalities concerned, a civil protection plan that lists the major disaster risks and determines risk reduction goals and how to attain them. In addition, municipalities are legally obliged to implement specific actions provided for in the civil protection plans and, for this reason, must prepare their own emergency management plans.

Quebec municipalities are therefore greatly involved in natural disaster risk management, but they can count on the technical and financial support of Quebec governments to meet their responsibilities [9, 10]. QDPS regional offices thus support municipalities in the preparation of their emergency management plans and in responding to weather-related disasters. Financial aid is also available to help municipalities build their prevention and preparedness capacities and recover from weather-related disasters (L.R.Q., C. S-2.3)).

## Theoretical Framework

We present here the study’s principal hypotheses and their background.

### Preparedness for Weather-related Disasters

Preparedness is the set of activities undertaken in advance of an incident that enables municipalities to respond to weather-related disasters and recover from them [11, 12]. Preparedness is an important component of disaster management policies as the effectiveness of interventions in emergency situations depends largely on its level [13, 14]. For example, municipalities that have established warning systems and plans for evacuating and sheltering victims during flooding will, in all likelihood, have a greater capacity to limit losses caused by this hazard than those municipalities that have no such systems and plans.

Publications on the management of weather-related disasters by municipalities are rare and conceptual studies predominate [15]. For this reason, the literature review of this study was not limited to the publications on the activities of municipalities in this domain but extended to public organizations in general, on the one hand, and to adaptation to climate change on the other. Available evidence suggests that several factors exert a significant influence on the preparation for weather-related disasters, such as risk exposure, availability of expertise and financial resources, and political incentives [13, 14, 16, 17, 18, 19, 20]. These factors can be grouped together under three categories, namely, the institutional framework, the risks of weather-related disasters and, finally, municipalities’ preparedness capacity.

#### Institutional Framework

The institutional framework provides the incentives, obligations and resources for local decision makers and is thus essential for understanding why municipalities take actions to protect the population and property from weather-related disasters. As mentioned above, the Civil Protection Act of Quebec delegates large responsibilities for emergency management to municipalities but, in return, requires them to prepare a civil protection plan identifying risk reduction objectives and defining the means to attain them.

The decentralisation shifts the accountability for the reduction of disaster risks to the local level and, for this reason, it is logical to consider the municipal decision-making process as a key factor in understanding the municipalities’ preparedness for and response to weather-related disasters. In this regard, the median-voter model postulates that, in a representative democracy such as that of Quebec municipalities, the supply of municipal services should be aligned with the preferences of the voter who is at the centre of the political spectrum [21]. The influence of the median voter on the outcomes of the decision-making process stems from the fact that each political coalition needs the support of this elector to win elections and to form the governing coalition. Studies using the median voter framework have shown that municipal expenditures are strongly correlated with the variables used as proxies for the median voter needs for municipal services and the capacity to afford their costs such as population size, property value and median household income [22, 23, 24].

Works on the politics of weather-related disasters also stress the importance of taking into consideration the electoral process. Indeed, studies on the US federal government indicate that the quality of the management of natural hazards has a positive correlation with the number of votes for the ruling parties [17, 18], and, presumably for this reason, political parties take into consideration voters’ needs in their natural disaster risk management policies [20]. In a well-functioning democracy, municipal elections are thus a strong political incentive that induces elected officials to commit to reducing the risk of weather-related disasters.

Decentralization is thus supposed to enhance the emergence of a civil protection service offer that is diversified and adapted to the needs of local communities [3]. To better understand this diversity, close attention must then be paid to factors explaining the demand for these services. As suggested by disaster management literature, the most important factors are the risk of weather-related disasters and preparedness capacity [13, 14, 16, 19].

#### The Risk of Weather-related Disasters

Weather-related disasters can result in death, injuries or serious diseases, damage to property, infrastructure or the environment, and financial losses due to the disruption of economic and social activities. The extent of these negative impacts is the result of the interaction between the risk of exposure and the vulnerability to extreme weather events [25].

Quebec municipalities are exposed to several weather-related hazards, notably periods of intense cold/heat, snow and freezing rain storms, other non-winter storms, and flooding or coastal submersion. The resulting damages increase in relation to the level of vulnerability, that is, municipalities’ socio-economic characteristics and physical environment that render them more or less likely to suffer losses following exposure to extreme weather phenomena [16, 25, 26, 27]. The risks of weather-related disasters are therefore high in vulnerable regions that are frequently exposed to such events.

Several studies suggest a positive relationship between the risk of weather-related disasters and the levels of preparedness [19, 28, 29, 30]. This positive relationship can be explained by the fact that the net return on investment in weather-related disaster risk reduction, generally expressed in terms of avoided losses, increases with the risk of weather-related disasters. When this risk is higher, taxpayers would be more willing to support weather-related disaster protection efforts and incur the related costs [31, 32, 33].

#### Municipalities’ Preparedness Capacity

In this study, weather-related disaster preparedness capacity of municipalities is defined as the ability to acquire the human, financial, material and information resources needed to implement an emergency management service responsible for evaluating the risks of weather-related disasters and reducing their impacts. This is a potential that is used to varying degrees by municipalities to build their capabilities to respond to extreme weather phenomena.

The available evidence indicates that there is a positive correlation between preparedness capacity–measured by variables such as budget, personnel and population served–and the level of preparedness for natural disasters, including weather hazards [28, 29, 30, 34, 35, 36]. At the international level, a negative correlation is found between casualties caused by natural hazards and the economic development level of countries, a relationship that may be explained by the fact that as a country develops its economy, it devotes greater resources to the management of natural hazard risks and, consequently, reduces its vulnerability [1, 37, 38].

### The Response to Weather-related Disasters

Municipalities’ response to weather-related disasters refers to activities undertaken immediately prior to and during impact or the acute phase of a weather hazard [39]. Quebec municipalities are therefore required to reduce losses caused by weather hazards by taking actions to protect population and properties, critical public infrastructure and guarantee the functioning of municipal services [40]. Their response includes planning and organizing the reaction to threatening events, such as the mobilization of emergency management personnel, and the interventions actually undertaken to protect the public, such as issuing weather warnings and orders to flee high flood risk areas.

Municipalities’ response to natural disasters is presumed to be a function of their level of preparedness, the risks of weather-related disasters and the quality of information that emergency management services have at the moment of organizing their response. The arguments regarding the relationship between preparedness and response have already been presented above. In the following paragraphs, emphasis will then be placed on the risk of weather-related disasters and quality of information.

Recent studies show that voters’ dissatisfaction with the party in power increases with the losses caused by the vagaries of nature [17, 18]. As the harm caused by weather hazards increases with the vulnerability level of municipalities, it can be inferred from these studies that elected officials of the most vulnerable municipalities, notably the mayors, would request emergency management services under their responsibility to respond vigorously to violent weather events in order to prevent losses and, as a result, avoid the risk of being blamed by voters for their inaction. A positive correlation is thus expected between the risk of weather-related disasters and the level of municipalities’ preparedness.

With regard to the quality of information, most countries have set up warning and advisory systems to inform the public and emergency management services about extreme weather events [39]. To be useful, information produced by these systems must be reliable and relevant [41]. Indeed, the aim of weather warnings is to provide information needed by users to accurately assess the risks and consequences of exposure to weather hazards and take adequate protective measures [42]. A high probability of false warnings or failure to detect hazards and issue corresponding alerts is likely to undermine the public trust in the warning system and, as a result, the use of the information provided [43]. Weather warnings should also be issued sufficiently in advance and updated regularly to give users enough lead time to take appropriate protective actions and adapt their response to the evolution of weather conditions [44]. Finally, warning messages must be written in language that is clear and simple so that users will understand it [45].

### Study’s Hypotheses

While several models for individual-level preparedness and responses to weather-related disasters have been published [46, 47], the same cannot be said for municipalities. This section thus proposes a model that depicts the principal relationships between the variables that, as was shown in the preceding sections, are presumed to have a significant influence on municipalities’ weather-related disaster preparedness and response.

**Fig 1:**
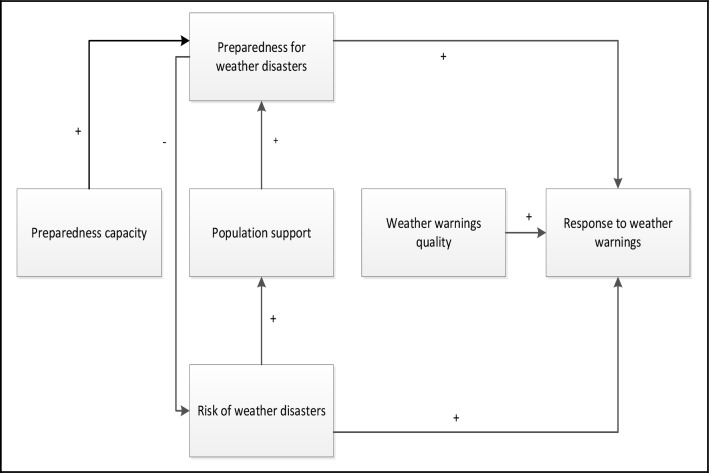
The determinants of weather-related disaster preparation and response.

Given that in Canadian municipalities mayors and council members are elected, it is assumed in the theoretical framework proposed in Fig 1 that the accountability of local decision makers to their citizens motivates them to develop a disaster management plan that matches the needs of the population. More specifically, elected officials are supposed to protect population from weather hazards, taking into account the risks of weather-related disasters and municipalities’ preparedness capacity. To achieve this goal, municipalities are expected to build their capability to respond to weather hazards and use it judiciously, particularly by taking into account the available information about threatening meteorological phenomena and the vulnerability of their communities.

In Fig 1, preparedness capacity is thus assumed to have a positive effect on the actual level of preparedness for weather-related disasters. Also, the risk of weather-related disasters is supposed to increase population support for the development of emergency management services and, in turn, this support translates into policies aimed at strengthening municipalities’ preparedness. Moreover, the municipalities’ preparedness is expected to have a negative association with risks of weather-related disasters. This hypothesis is based on the assumption that weather hazards have less impact on municipalities that have managed to develop their disaster risk management services so as to be able to intervene effectively when their territory is threatened.

In addition, the risk of weather-related disasters is supposed to be positively correlated with the response to weather warnings. The reason is that, if an issued warning was accurate and the municipality effectively hit by the threatening weather event, then the financial, social and political costs of ignoring the warning and refusing to take the necessary steps to protect the population would increase with the level of the municipality’s vulnerability.

Municipalities with a high level of preparedness are also supposed to have the structures, skills and resources that help them to take the necessary measures to fight weather hazards. Finally, it is presumed that disaster management officials are more likely to use information on threatening weather phenomena when this information is perceived as reliable and relevant and issued in timely fashion. In sum, we assume that once a weather warning is issued, the response of the municipalities will be the result of a decision making process assessing 1) the probability of exposure to the weather hazard, 2) the consequences of exposure and 3) the capacity to cope with it [48]. In the model, these three components of the decision making process are respectively represented by the quality of weather warnings, the risk of weather-related disasters and the level of preparedness.

The model is based on the assumption that local elected officials are motivated by their institutional framework to develop a civil protection service that is well adapted to population needs. However, extreme weather events remain rare and difficult to foresee, so the population may underestimate the losses that could be incurred, an attitude that would result in marginalizing the emergency management issue on the municipal political agenda [14, 36, 49, 50]. In addition, citizens are not often well-informed about decisions made by their representatives. The latter may then adopt natural disaster management policies benefitting certain interest groups to the detriment of the general population [49]. The underestimation of risks in conjunction with the lack of transparency will thus distort the implementation of an effective weather-related disaster risk management plan.

## Methodology

### Data Sources

Two sources have been used to compile data on municipalities’ preparedness for weather-related disasters and response to weather warnings, namely, an online survey and administrative data. As is the case of similar previous studies, the survey data were obtained from municipal emergency management coordinators [51]. These stakeholders play a key role in the preparation and implementation of municipal emergency management plans, and, as a result, they represent a valuable source of information.

An online survey was thus used to gather data on the perceived risks of weather-related disasters, municipalities’ level of preparedness, the quality of weather warnings and response to these warnings, among other things. The questions on weather warnings relate to alerts received by municipalities from the regional offices of the QDPS over the twelve months preceding the survey. Warnings issued by the regional offices were taken into consideration because these bodies are responsible for monitoring extreme weather events and informing municipal emergency management coordinators.

The authors of this study designed the first version of the questionnaire and sent it to the regional directors of the QDPS to collect their comments and suggestions. The proposals of these experts in emergency management were then taken into account when drafting the final version. As for administering the survey, we had no direct access to email addresses of the municipalities’ emergency management coordinators. So, after consulting with representatives of the QDPS, the regional directors of this department were requested to send invitations to participate in the survey to the municipal coordinators of emergency management in their respective administrative regions.

The survey of the coordinators of municipal emergency management took place between April 15 and May 31, 2014. Given that an online survey was used in this study, participants were required to sign an electronic informed consent form before completing the survey. This procedure for obtaining informed consent, as well as the other elements of the design of this study, was approved by the ethics committee of the *Institut National de la Recherche Scientifique*.

After three reminders, 233 emergency management coordinators responded to the survey. Given that the questionnaire was administered by third parties, it was not possible to obtain accurate information about the number of persons who received the invitation to participate in the study. Consequently, we are not in a position to determine the exact response rate. However, according to the data from the QDPS, out of 1,117 municipalities in Quebec, 1,029 currently have an emergency management coordinator. Assuming that all coordinators received invitations to participate in the survey, the response rate would be at least equal to 22.6%.

The response rate may raise questions about the representativeness of the study data. The analysis of municipalities’ characteristics indicates that the size of the population of the respondent municipalities is higher than the average of Quebec municipalities (14,221 versus 7,362). Large municipalities are thus over-represented in the database of this study. The overrepresentation of large municipalities could be explained in part by the fact that many small municipalities do not have an emergency management plan. Quebec’s Civil Protection Act indeed authorizes them to delegate this function to other municipalities or to regional authorities such as the MRC. The analysis also reveals slight differences between respondents’ and non-respondents’ municipalities regarding the value of the standardized property value per capita ($91,386 versus $90,301) and the median household income ($46,787 versus $45,044), but these differences are not statistically significant.

In addition to the online survey data, available official statistics were used in this study. Thus, the data on the standardized property values were extracted from the municipalities’ 2011 financial profile found on the website of the Quebec Ministry of Municipal Affairs and Land Occupancy. The data on population were obtained from Statistics Canada, based on the 2011 population census and national household survey.

### Study Variables

Risk of Weather-related Disasters. In this study, emphasis was put on the most frequent weather-related hazards in Canada, namely cold spells, snowstorms, freezing rain storms, heavy rains (and their consequences including flooding), non-winter storms, heat waves, and smog episodes.

Objective methods exist to measure the risks of weather-related disasters, such as the inventory of the number of exposures to extreme weather events and the estimation of resulting damages. These types of data, however, are not systematically available in the case of Quebec municipalities. The perceptions of municipal emergency management coordinators have therefore been used to measure the risks of the extreme weather events. Specifically, the respondents were to rate, on a scale of 1 to 5 (1: very low and 5: very high) to what extent each of the extreme events listed above represents a danger for the population of their municipalities.

#### Preparedness for Weather-related Disasters

Three factors were taken into account in the analysis of the level of weather-related disaster preparedness, namely the availability of personnel, equipment and installations, and financial resources. Municipal emergency management coordinators had to indicate, on a 4-level scale, to what extent these resources are sufficient or insufficient to protect the population from weather-related disasters. The same scale was also used to see if, in general, the level of municipal preparedness was sufficient or insufficient.

#### Population Support for Weather-related Disaster Risk Management

Two items were used to measure the emergency management coordinators’ perception of population’s support for policies and programs aimed at reducing the risk of weather-related disasters. The first item deals with the population’s level of concern with regard to the risks of weather-related disasters. A scale of 1 to 4 was used for this purpose, with 1 referring to a not-at-all important concern and 4 to a very important concern. The second item deals with the population’s support for initiatives to strengthen municipality’s preparedness to extreme weather events. The level of support was measured on a scale of 1 to 5 (from 1: very weak to 5: very strong).

#### The Quality of Weather Warnings

Four dimensions were taken into account in the analysis of the quality of weather warnings. The first dimension deals with the accuracy of weather warnings, namely, the probability that the forecasts of extreme weather events do really occur, or in other words, are not false alarms. The accuracy of warnings is measured on a scale of 1 to 4, as follows: 1 indicates that the warnings are never accurate, and 4 indicates that the warnings are always accurate. The second dimension deals with the relevance of issuing weather warnings. The same scale was used to ascertain if the warnings were, or were not, related to extreme weather events representing a real danger to the municipalities (1: never a real danger and 4: always).

The third dimension measures the usefulness of information on weather hazards found in the warning messages, such as time, length and intensity of exposure to the weather hazards. Information usefulness was evaluated on a scale of 1 to 4, with 1: not useful at all, and 4: very useful. And the last dimension aims to find out to what extent weather warnings are issued far enough in advance to permit municipalities to prepare their responses. A scale of 1 to 4 was used for this purpose (1: the lead time of the warnings is very insufficient to 4: very sufficient).

#### Response to Weather Warnings

After consulting the Quebec Government’s guide on the preparation of municipalities’ response to natural and technological hazards (*Guide sur la préparation de la réponse des municipalités aux sinistres)* [52], the following four decisions of the municipal emergency management coordinators were retained to measure their response to weather warnings: 1) inform the managers of their municipalities who are concerned by emergency management issues of weather warnings, 2) assemble these managers to prepare the response of the municipality, 3) inform the municipality’s partners of the weather warnings, and, finally, 4) mobilize the personnel of the emergency management service so that they are ready to intervene. A scale of 1 to 4 was used to measure the frequency of each of these decisions (1: never to 4: always).

#### Preparedness Capacity

In this study, two variables were used as proxies to the municipality’s weather-related disaster preparedness capacity. They are the standardized property value and population size. The standardized property value measures municipalities’ fiscal capacity to pay the costs of creating and operating emergency management services. Property tax in Quebec yields approximatively 50% of municipal revenues [53]. Given that grants to municipalities for civil protection in 2014 represented less than 10% of the cost of these services, property value constitutes the principal source of funding weather-related disaster risk management and therefore, is an adequate proxy to municipalities’ fiscal capacity (Data on Lac-Mégantic were not included in computing the share of grants as this city was the theatre of a tragic petrol train derailment, explosion and large scale downtown fire that caused 47 deaths and called for a substantial financial aid from the Quebec government).

Population size is also related to municipalities’ capacity to pay for emergency management services. In fact, implementing such services involves significant fixed costs like the construction of buildings, purchasing equipment and hiring permanent staff. Because of economies of scale, the cost per capita could be a decreasing function of population size. Large and wealthy municipalities would therefore have more means to provide services to tackle weather-related disasters than small and less affluent municipalities.

### Methods of Data Analysis

With the exception of population size and standardized property value that are continuous variables, this study’s variables are measured on scales of four or five levels, depending on the case. As suggested by Carifio and Perla [54] we thus used percentages to describe the responses to individual items of the questionnaire. We have also constructed composite indices by summing responses to the items belonging to the same theoretical construct and then used parametric statistics to analyze them. In addition to enabling the use of parametric statistics, the construction of composite indices is appropriate for the purposes of this study as the variables of the theoretical model are multidimensional constructs and, therefore, several survey items should be used to measure them. The description of the items that make up each index as well as the Cronbach’s alpha measuring the internal consistency of the indices are presented in Tables 1 to 4. Depending on the variables, the Cronbach’s alpha is between 0.64 and 0.87, which are generally considered as satisfactory levels [55].

**Table 1:** Risks of weather-related disasters.

Measurement scale	Very low	Low	Moderate	High	Very high
Thunder storms	0.9%	8.7%	33.3%	38.1%	16.8%
Snow storms	1.3%	10.8%	34.0%	39.7%	14.2%
Heavy rain	2.6%	11.6%	35.8%	37.1%	12.9%
Floods	10.3%	22.4%	28%	22.4%	16.8%
Ice storms	0.9%	12.7%	39.7%	23.7%	14%
Cold spells	5.2%	20.8%	41.1%	24.2%	8.7%
Heat waves	15.2%	31.2%	35. 5%	15.2%	3%
Smog episodes	56.5%	29.6%	10.9%	0.3%	0.04%
*Cronbach’s alpha*: *0*.*79*					

**Table 2:** Municipalities preparedness for weather hazards.

Measurement scale	Very insufficient	Insufficient	Sufficient	Very sufficient
Staff	3.9%	28.13%	59.31%	8.7%
Equipment & installations	6.5%	39%	48.5%	6.1%
Budget	15.2%	47.2%	33.3%	4.3%
Preparedness level	4.3%	26%	64.1%	5.6%
*Cronbach’s alpha*: *0*.*87*				

**Table 3:** The quality of weather warnings.

Measurement scale	1	2	3	4
Accuracy (1: rarely, 4 : always)	0.4%	9.9%	57.9%	31.7%
Relevance (1: rarely, 4 : always)	3.2%	18.5%	51.6%	26.7%
Usefulness (1: not at all useful, 4:very useful)	0%	7.2%	52.9%	39.8%
Lead time (1:very insufficient, 4 :very sufficient)	0%	5%	71.9%	23.07%
*Cronbach’s alpha*: *0*.*64*				

**Table 4:** The response to weather warnings.

Measurement scale	Never	Rarely	Often	Always
Inform managers concerned by emergency management issues	6.4%	15.9%	25.5%	52.3%
Assemble managers concerned by emergency management issues	21.1%	49.5%	19.7%	9.6%
Mobilize personnel	11.4%	40%	34.5%	14.1%
Inform municipalities’ partners	46.8%	38.4%	12.3%	2.7%
*Cronbach’s alpha*: *0*.*73*				

In this study structural equation modelling was used to test the validity of the hypotheses related to the factors that in Fig 1 are presumed to affect the levels of preparedness for weather-related disasters and response to weather warnings. This technique is well suited to disentangle the complex set of relationships underlying the phenomena studied, notably by accounting for the endogeneity between the explanatory variables and for the situation in which the effect of some variables is mediated by other variables [56].

## Study Results

### Weather-related Disaster Preparedness and Response

Descriptive statistics of survey data are presented in Tables 1 to 5. Table 1 is about emergency management coordinators’ risks perception of eight hydrometeorological hazards. The risk perception reflects in general the reality of the Canadian climate as the majority of respondents believe that thunder storms (49.9%), snow storms (53.9%) and heavy rain (50%) represent a high danger for the population while only a small portion of them think such is the case with regard to heat waves (18.2%) and smog episodes (0.34%).

**Table 5:** Population support for weather disaster management.

Measurement scale	1	2	3	4	5
Population concern (1: not at all important, 4: very important)	5.2%	35.3%	48.3%	11.2%	NA
Population support (1: very weak, 5: very strong)	3%	23.8%	46.3%	22%	4.8%
***Cronbach’s alpha*: *0*.*68***					

The data of Table 2 indicate that 69.7% of respondents believe their municipalities have a sufficient or very sufficient level of preparedness for weather-related disasters. The majority also think there are sufficient staff (68.01%), equipment and installations (54.6%) to deal with extreme weather events. On the other hand, more than half (62.4%) complain about the lack of financial resources.

With regard to the quality of weather warnings, 95% of municipalities received alerts from the regional directions of QDPS during the year preceding the survey. The statistics of Table 3 show that a very strong majority of respondents think that the warnings are accurate (89.6%), relevant (78.3%) and useful (92.7%). Furthermore, 94.97% believe that the current lead time for weather warnings is sufficient.

According to data of Table 4 on response to weather warnings, 77.8% of emergency management coordinators often or very often inform senior officials of their municipalities who are concerned by emergency management issues of weather warnings but only a minority (29.3%) of them regularly convene these stakeholders to plan the response to weather warnings. When they receive weather alerts, almost half of the respondents (48.6%) often or very often mobilize the emergency management personnel of their municipalities to be ready to intervene in case of a disaster. However, only 15% of the coordinators often or very often relay weather warnings to the partners of the municipalities that are involved in the management of weather-related disasters. The dissemination of information within the municipalities of the respondents and the mobilization of civil protection personnel thus appear to be the most frequent responses to weather warnings.

Finally, 59.5% of respondents believe that the risk of weather-related disasters is an important or very important concern for the population and that, in most cases, the population shows moderate (46.3%), strong (22%) or very strong (4.8%) support for projects to improve the level of municipal preparedness for weather hazards (Table 5).

### The Determinants of Weather-related Disaster Preparedness and Response to Weather Warnings

**Fig 2:**
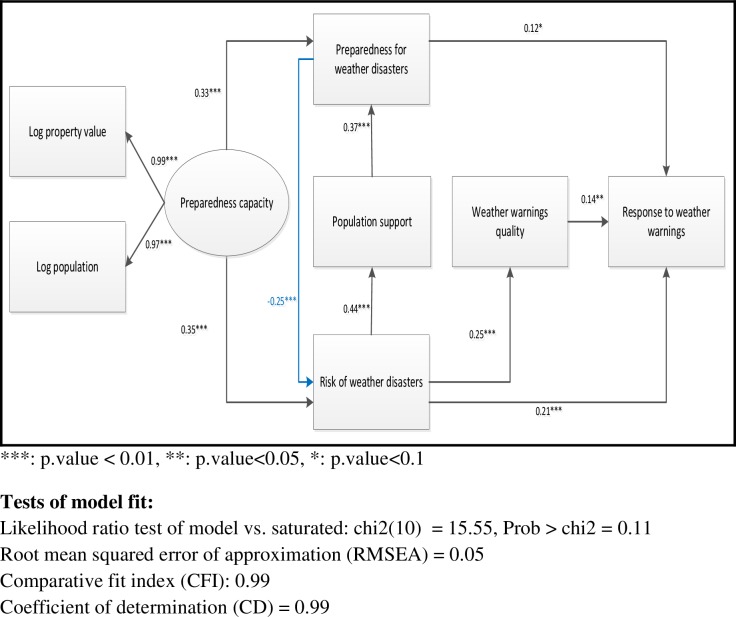
Estimation of the parameters of the model.

This section is devoted to the results of testing the relationship between the variables of the theoretical model presented in Fig 1. In comparison to this figure, two links were added to the empirical model. Preparedness capacity was linked to the risk of weather-related disasters, which in turn was connected to the quality of weather warnings. Judging by the goodness-of-fit statistics commonly used to evaluate structural equation models (see notes at the bottom of Fig 2), the addition of these links has greatly improved the capacity of the model to fit the data. This improvement can be explained by the fact that the new links measure real relationships between the variables in question. As we will discuss later, previous studies suggests that the variables used in this article to measure preparedness capacity are also correlated with the risk of weather-related disasters. Concerning the second new link, municipalities most at risk tend to have acute needs for information about weather hazards and, as a result, are more likely to have positive appraisal of the usefulness of weather warnings.

Of the 233 municipalities that responded to the survey, 32 were excluded from the analysis either because they did not receive weather warnings during the year preceding the survey (n = 13) or because of missing data (n = 19). The analysis therefore focused on 201 municipalities. The logarithms of the population and the standardized property value were used in the analysis because the distribution of these two variables is highly concentrated toward the left (positive skewness).

The model, whose results are presented in Fig 2, was estimated using the structural-equations module of the STATA software.

To facilitate interpretation of results, standardized regression coefficients are reported in the model. A standardized β coefficient signifies that a one standard deviation change in the predictor variable results in a β standard deviation change in the dependent variable.

The analysis indicates that the preparedness capacity has a positive association with the municipalities’ preparedness level for weather hazards (β = 0.33). As noted in the theoretical framework, municipalities with a high capacity seem to be better prepared for weather hazards. Preparedness capacity also has a positive relationship with the risk of weather-related disasters (β = 0.35). To understand this relationship that at first seems to be counter-intuitive, it should be noted that, in this study, preparedness capacity is a latent variable measured by indicators that are closely related to the size of the municipality, namely population size and standardized property value. Large cities are thus supposed to have a greater capacity of preparedness for weather-related disasters but, at the same time, they are particularly vulnerable to these disasters because of the high concentration of people and wealth, as well as the presence of vital and interdependent infrastructure systems [33, 57].

In addition, the analysis shows that the risk of weather-related disasters has a positive association with population support to reduce these risks (β = 0.44), suggesting that the population’s support for the policies of weather-related disaster risk reduction increases with the risk level, or at least its perception. Population support, in turn, has a positive impact on the level of preparedness (β = 0.37). The analysis also suggests that the enhancement of the level of preparedness reduces the risk of weather-related disasters (β = -0.25).

These results also indicate that municipalities’ response to weather warnings increases with the risk of weather-related disasters (β = 0.21), the quality of weather warnings (β = 0.14) and, to a lesser extent, the municipalities’ preparedness level (β = 0.12). Lastly, municipalities at higher risk of weather-related disasters have a more positive assessment of the quality of weather warnings (β = 0.25). This positive relationship may be explained by the fact that the need for weather warnings increases with the probability of being harmed by weather-related disasters. Taken together, these findings lend substantial support for the hypotheses of this study according to which 1) the level of preparedness for weather-related disasters increases with preparedness capacity, weather-related disasters’ risks and population’s support for efforts to contain these risks, and 2) the levels of preparedness and risks and the quality of weather warnings have positive effects on the responses to weather warnings.

## Discussion and Conclusion

Disaster management frameworks assign local governments a prominent role in the reduction of the risks caused by weather hazards [2]. The reason is that decentralization is supposed to increase the accountability, transparency and responsiveness of local decision makers and, consequently, improve the management of public services whose benefits mainly accrue to the households residing in the boundaries of the municipalities providing them [3]. The delegation of powers to municipalities thus holds the promise for improving the management of weather-related disasters but, at the same time, carries the risk of some municipalities suffering from the under-provision of these services due to lack of political incentives and weakness of fiscal and administrative capacities [1, 4]. Despite the importance of weather-related disasters management responsibilities delegated to municipalities and the concerns this transfer of powers may raise, few quantitative studies have dealt with this issue.

To fill this gap, this article presented the results of a quantitative study on the extent and determinants of Quebec municipalities’ preparedness for weather-related disasters and response to weather warnings. These results indicate that most municipalities have a satisfactory preparedness level for extreme weather events. However, municipalities with a weak preparedness capacity, i.e. small and less affluent ones, are less prepared for weather disasters. This relationship raises concerns about the delegation of weather-related disaster risk management to municipalities since efficiency gains from decentralization could be achieved at the price of increased inequity of protection from extreme weather events across communities. On this matter, the Government of Quebec has introduced financial and technical support for municipalities such as the programs to compensate for losses caused by extreme weather events and technical assistance provided by the QDPS regional offices. It has also implemented unconditional grants programs to provide financial support to less affluent municipalities and conditional infrastructure grants programs to cover the costs of infrastructure projects, including projects designed to strengthen the capacity of municipalities to prevent and respond to weather-related disasters [23]. However, this study shows that some disparities remain across municipalities and need to be addressed in the future.

This study also shows that the risk of weather-related disasters has a positive impact on municipalities’ preparedness level and that this effect is channelled through the population’s support for efforts to mitigate weather hazards. The relationships between these variables bring to the attention two issues that may be of great interest to scholars of emergency management. First, the literature on decentralization in general suggests that the fit between population’s needs for protection from weather-related disasters and the level of municipal preparedness depends on the existence of an institutional framework that enables citizens to hold elected officials accountable for their performance [15]. In Quebec, general elections that are held every four years and monthly public municipal councils provide such a mechanism. In countries with weak democratic institutions or where decentralization is merely administrative rather than political, citizens have less control over and interaction with local decision makers, a situation that encourages the latter to be less responsive to the needs of the former.

The second concern is related to the “natural” tendency of the population to underestimate the risk of weather-related disasters [14, 36]. The correlation between the risk level of weather-related disasters and population support found in this study implies that when this information bias is significant, it results in municipalities providing lower levels of protection from weather-related disasters to their citizens. Public campaigns to raise awareness of the real risk levels help to build political support for emergency management policies and, consequently, to improve the municipalities’ level of preparedness.

With regard to responses to weather warnings, study data indicate that most emergency management coordinators prepare to intervene when they receive weather warnings from the QDPS regional offices. This positive reaction can be, to some extent, attributed to the quality of the warnings. In fact, a large majority of respondents have a very positive assessment of the reliability, relevance and usefulness of weather warnings. As shown by the structural equation model, these characteristics are positively correlated to the responses to weather warnings. Given this positive correlation, continuous improvement of the quality of weather warnings is needed to help emergency management services respond more effectively to weather hazards. This could involve, for instance, the improvement of weather forecast accuracy and the establishment of weather warning thresholds that take local vulnerabilities into consideration. Finally, the analysis indicates that attention to warnings increases with the perceived risk of weather-related disasters and municipal preparedness level.

While this study contributes to research on municipal disaster risk management by providing new evidence on the levels and drivers of municipalities’ preparedness for weather hazards and response to weather warnings, it has some limitations that deserve to be made explicit. First, several constructs are measured by the respondents’ perceptions. This is not a weakness in itself since, as shown by cognitive models, perceptions are among the main determinants of behaviour [58]. However, it is desirable to use different sources of information in order to mitigate common measurement biases that arise when, for instance, respondents who report that their organizations have a high level of preparedness may be more likely to overestimate the population support for their activities. Also, the effects of emergency management coordinators characteristics (age, gender, seniority, etc.) on the level of preparedness and response were not tested because data on these characteristics were not collected. To this must be added that the cross sectional design of this study raises concerns on the direction of causality between the variables of the model. The use of longitudinal data will help to overcome this problem in the future. Finally, this study shows that the risk of weather-related disasters, preparedness and quality of weather warnings have positive effects on municipalities’ response to weather warnings. But it is difficult to infer from this analysis that these factors lead municipalities to improve their interventions and, as a result, diminish the costs of weather-related disasters. Further research is therefore needed to explore these issues.

## Supporting Information

S1 Municipalities (DTA)

S2 Municipalities (DO)
